# Letter from La.

**Published:** 1854-04

**Authors:** George J. Huey

**Affiliations:** Atchafalay Parish, St. Landry, La.


*Letter from La.*

[The following extract from a private letter from our much respected friend Dr. Huey, will interest many of our readers.]

ATCHAFALAY PARISH, ST. LANDRY, LA.
Jan. 25. 1854.

DR. HERRICK:—The diseases of this section are such generally as will be found most commonly throughout the great valley of the Mississippi. Itermittents, Remittents, Typhoid, Pneumonia, Rheumatism, Dysentery, Diarrhœa in a common way. This summer has formed an exception, in the way of Epidemic Yellow Fever, a few cases of which was encountered in my neighborhood, at least it was the same disease which prevailed throughout the South this summer and fall, and was most generally pronounced Yellow Fever, though it was denied as being Yellow Fever by some of the most eminent Physicians of New Orleans, and other localities; for example Dr. Warren Stone, of New Orleans, said it was a combination of Yellow Fever and African Plague. I have not seen anything from his pen regarding it, but that is the general report respecting his opinion concerning it. The symptoms of the fever which prevailed here, and which I attended and treated (some seven or eight in number) were—first, an indisposition, slight for a longer or shorter time, generally 12 or 24 hours—when would ensue severe pain in the head and back, more so than is generally found in remittent fever, great suffusion of eyes, which were dull and heavy looking, tongue red and broad with a white mucus in the middle in some cases—and in others bilious coating in the middle. Bowels costive, great heat of surface generally; nausea and vomiting—this was *most generally* indeed a troublesome symptom, and the character of the matter vomited was bilious. Pulse—from 110 to 120 in the minute, generally full, these symptoms were high for 3 or 4 days, when they would assume more of the typhoid type. Another prominent symptom was bleeding from the nose and mouth, and in one case, continued oozing of blood from the gums lasting 9 days.

The character of the discharges from the bowels, showed ıl formed its principal ingredient. The treatment was at first to use the warm mustard bath (that is foot-bath); when they would be wiped dry, placed in bed, and given from 10 to 20 grains blue pils or, Cal. gr. 10 Dov. gr 8 and at the expiration of 8 hours, a large dose of Caster Oil and Spirits Turpentine, to insure free action on Bowels. For sick stomach. mustard plasters were applied, in connection with soda powders given internally; and after the bowels were freed and the sick stomach measurably ceased, I gave a diaphoretic of Nitre and Dig., most generally. In one case, that of a stout young man, after the operation of the cathartic as above, I commenced with the Quinine in large doses, (on the evening of the second day of the fever) 60 grains in 24 hours, at intervals of 8 hours 20 grains, till he he had taken in the 48 hours 120 grains, which was followed by profuse diaphoresis, cessation of fever, and convalescence on the 5th day. In the other cases I did not use the Quinine to the same extent—but relied more on the diaphoretic proper—using an *expectant* treatment. In the case of bleeding as mentioned, in conjunction with the remedy mentioned, used the chlorate of potash 40 grains to 3 oz. water, in table spoon doses once in 12 hours. The potash was used to supply oxygen the blood, which was considered deficient in that particular. In this case there was in connection with the symptoms stated *great* lessness during the whole time, at least till convalescence ensued.

This is a rough sketch of the disease, as was seen here, and I suppose you would like to hear, whether they got well or died.—With pleasure I can say they all recovered. Now, whether it was owing, in any great degree, to my treatment or not, I cannot tell as Prof. Fitch of R. Med. College used to tell us, our patients may get well, in *spite* of our treatment, and I confess this has often occurred to my mind, and has perplexed me much.

I have a case to relate to you, of a different character. A young lady now 18 years of age, in the year 1850, was taken with Parotitis on both sides which continued in the usual way, for 5 or 6 days, when she began to get better. At this juncture she imprudently exposed herself in a damp gallery, and was taken with fever the swelling returned, and extended to the clavicles and even behind the ears—in fine the whole of the throat and jaws swelled prodigiously, and interrupted the breathing very much—and in connection with this and the fever, vomiting came on one evening during the efforts of which she became partially deaf, and the next day, she became totally deaf, and has remained deaf ever since. The fever continued for some time afterwards, and she had in common terms, what is called a long spell of sickness. She was taken in the summer and did not *go about* for 8 months. Ever since this spell, her health, contrary to all expectations, has been tolerably good—and she retains to a remarkable degree, the power of speech, and that with good modulation and intonation of voice. One unacquainted with her, on hearing her talk, would hardly suppose her to be deficient in hearing. She is lively in disposition, and intelligent. The glands around the throat are ar dened, and the opening of the Eustachean tube within the mouth seems to present some degree of contraction. The tube is not entirely closed, at least was not a year ago. There is little or no secretion from the external ear, and there has never been any running of any kind, indicating anything like disorganization of the parts; but the ringing of the largest bell, over her head, she cannot hear. The firing of a cannon, or report of a gun, makes an impression through the sense of touch. Now the small bones of the ear may be affected, Paralysis of the auditory nerve in connection with the partial occlusion of the Eustachean tube may suffice to produce the result, I suppose, but to see the cause certainly is an object of inquiry. She is frequently troubled now with a roaring or rumbling in her ears—and I tried the effect of Quinine on her, and it produces a ringing in her ears—as she expresses it.

Your Friend,

GEORGE J. HUEY.

